# Raman spectroscopy as a tool to investigate the structure and electronic properties of carbon-atom wires

**DOI:** 10.3762/bjnano.6.49

**Published:** 2015-02-17

**Authors:** Alberto Milani, Matteo Tommasini, Valeria Russo, Andrea Li Bassi, Andrea Lucotti, Franco Cataldo, Carlo S Casari

**Affiliations:** 1 Department of Chemistry, Materials and Chemical Engineering “G. Natta”, Politecnico di Milano, Piazza Leonardo da Vinci 32, 20133 Milano, Italy; 2 NEMAS – Center for NanoEngineered Materials and Surfaces, Politecnico di Milano, via Ponzio 34/3, 20133 Milano, Italy; 3 Department of Energy, Politecnico di Milano, via Ponzio 34/3, 20133 Milano, Italy; 4 Dipartimento di Scienze Ecologiche e Biologiche, Università della Tuscia, Via Camillo de Lellis, Viterbo, Italy; 5 Actinium Chemical Research srl, Via Casilina 1626A, 00133 Roma, Italy

**Keywords:** carbon nanostructures, cumulenes, polyynes, Raman spectroscopy, sp-hybridized carbon systems

## Abstract

Graphene, nanotubes and other carbon nanostructures have shown potential as candidates for advanced technological applications due to the different coordination of carbon atoms and to the possibility of π-conjugation. In this context, atomic-scale wires comprised of sp-hybridized carbon atoms represent ideal 1D systems to potentially downscale devices to the atomic level. Carbon-atom wires (CAWs) can be arranged in two possible structures: a sequence of double bonds (cumulenes), resulting in a 1D metal, or an alternating sequence of single–triple bonds (polyynes), expected to show semiconducting properties. The electronic and optical properties of CAWs can be finely tuned by controlling the wire length (i.e., the number of carbon atoms) and the type of termination (e.g., atom, molecular group or nanostructure). Although linear, sp-hybridized carbon systems are still considered elusive and unstable materials, a number of nanostructures consisting of sp-carbon wires have been produced and characterized to date. In this short review, we present the main CAW synthesis techniques and stabilization strategies and we discuss the current status of the understanding of their structural, electronic and vibrational properties with particular attention to how these properties are related to one another. We focus on the use of vibrational spectroscopy to provide information on the structural and electronic properties of the system (e.g., determination of wire length). Moreover, by employing Raman spectroscopy and surface enhanced Raman scattering in combination with the support of first principles calculations, we show that a detailed understanding of the charge transfer between CAWs and metal nanoparticles may open the possibility to tune the electronic structure from alternating to equalized bonds.

## Review

### Introduction

Over the last decades carbon nanostructures have been widely investigated for their unique properties and for their potential technological applications [1]. For instance, single wall carbon nanotubes represent quasi-1D systems whose electronic properties are strongly related to the nanotube structure (i.e., chirality), while graphene is a 2D system with appealing electronic and optical properties [2–4]. In addition to structures based on sp^2^ hybridization of carbon atoms, sp-hybridized carbon-atom wires (CAWs) are intriguing systems with structure-, length- and termination-dependent properties [5]. Similar to graphene (which today is considered the ultimate 2D system (1-atom-thick)), CAWs represent a true 1D system (1-atom-large) which can display either semiconducting or metallic properties due to the conjugation and electron–phonon coupling effects of their delocalized π electrons.

In addition to many examples in organic chemistry, the occurrence of sp-hybridized carbon has been observed in many carbon-based materials and structures, embedded in cold gas matrices, in free carbon clusters in the gas phase, as pure sp-sp^2^ systems, in liquids, inside carbon nanotubes and connecting graphene sheets [5–13]. The research on sp carbon dates back to the last century when the carbon community was in search of a new carbon allotrope based on linear carbon. The first papers claiming observations of sp-hybridized carbon as a new allotrope (named carbyne, and the mineral form was called chaoite) date back to the sixties by Kudryavtsev and co-workers [14], by El Goresy and Donnay [15] and by Whittaker [16–17]. Criticism on the interpretation of these results was raised in the eighties by Smith and Buseck and were the objects of debate [18–20]. In the same period the search for linear carbon in interstellar medium for astrophysics studies drove the discovery of fullerenes by Kroto, Smalley and Curl, as reported in the Nobel lecture by Kroto [21]. Even though a new allotrope based on sp carbon has still yet to be found, sp-hybridized carbon nanostructures (or large molecules) in the form of linear atomic wires can be now produced and investigated. Great interest has been shown in the theoretical prediction of the electronic and transport properties of carbon wires connected to metal electrodes and to other carbon nanostructures such as graphene and nanotubes, while detailed experimental work is still needed to unveil the structure and properties of these systems.

Raman spectroscopy is a powerful tool for the characterization of carbon materials and nanostructures due to its sensitivity to the vibration of C–C bonds. For instance, strong electron–phonon coupling and resonance effects allow for the measurement of single carbon nanostructures and together with confinement effects, provides information on their structure, hybridization state, defects, presence of functionalization and/or doping, and can even quantify the nanotube chirality, the number of layers and the edge structure in graphene [22–23].

In this review we discuss how Raman spectroscopy can be utilized to obtain a wealth of information on the structure of CAWs including length, stability behavior and electronic structure changes induced by charge transfer effects. In particular, for different CAWs, the results of a combined standard Raman spectroscopy and surface enhanced Raman spectroscopy investigation at different excitation wavelengths with the support of first principles calculations will be reviewed. We begin by discussing the structure of ideal and as-synthesized CAWs with particular focus on π-conjugation effects and the change in electronic properties as a result of the wire length and termination. Then we review the various CAWs synthesis techniques and strategies to improve stability. Finally we present Raman and SERS characterizations of selected CAW systems.

### Structure of carbon-atom wires

**Figure 1:**
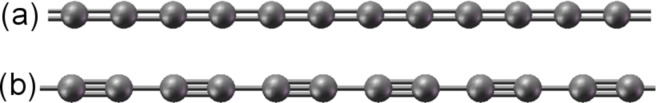
Schematic structures of infinite, linear, sp-carbon wires: (a) equalized wire with all double bonds (cumulene) and (b) alternating single–triple bond wire structure (polyyne).

The ideal model of sp-hybridized carbon wires is an infinite chain comprised of two different geometric arrangements of atoms, as depicted in Figure 1. One possibility is a sequence of double bonds in a completely equalized geometry (also called cumulene), and the other is a series of alternating triple and single bonds in a dimerized geometry (also called polyyne). The two configurations are physically related by stability issues since the 1D atomic equalized structures tend to change into the alternating triple–single bond structures to reach a minimum energy configuration (i.e., due to the onset of a Peierls distortion). Such structural change has a direct effect on the electronic properties.

Infinite cumulenes have one atom per unit cell, providing one electron from each 2p*_z_* orbital, thus forming a half-filled band of a 1D metal. As a consequence of Peierls distortion (driven by electron–phonon coupling and dimerization of the structure), an energy gap opens and the metallic character of cumulenes changes into the semiconducting behavior of polyynes, which corresponds to a lower energy of the ideal sp-carbon chain. Along this metal-to-semiconductor transition the vibrational properties are strongly modified. One of the major effects is the appearance of an optical phonon branch, which is otherwise absent in an equalized, monoatomic, infinite chain, such as the ideal cumulene.

Moving from ideal to real, as-synthesized structures, finite length effects and termination of the chain play a fundamental role. The end groups can affect the overall configuration and their effect becomes more significant with decreasing wire length. The control of the electronic properties such as the band gap and the conducting character by tuning the wire structure may open new opportunities for the realization of nanoscale cables and devices, as demonstrated by theoretical predictions [24–25] however still not experimentally demonstrated. Indeed, this possibility exploits the connection between the molecular structure, the electronic properties and the vibrational properties, which is well documented for all π-conjugated carbon systems [26–29]. The infinite wire model affords a reliable interpretation of the experimental data, portraying the main trends observed in both the electronic and vibrational features, and offering a unified framework for the analysis of the different linear carbon chains synthetized to date. Indeed, many works [29–35] have offered a detailed theoretical interpretation of the relation between the chain structure, band gap and Raman activity of the infinite chain, thus fostering the interpretation of the behavior of the existing finite length carbon chains. However, this approach also has some limitations due to non-negligible end effects. Such limitations may require the relaxation of the assumption of an infinite, atomic chain for the detailed discussion of real, finite-length systems, where the Peierls distortion effect, the stability of cumulenic versus polyynic chains and the vibrational structure (i.e., IR and Raman signals) must be considered.

**Figure 2:**
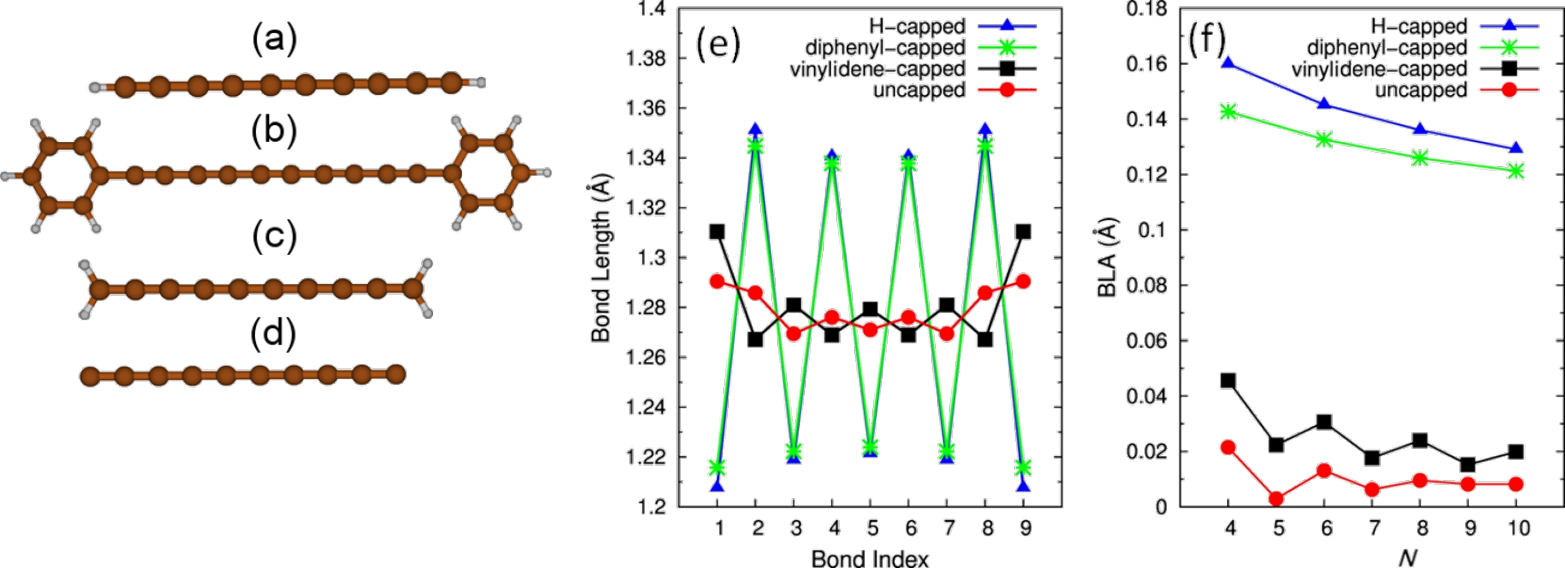
(a–d) carbon-atom wires with different terminations: hydrogen-capped (a), phenyl-capped (b), vinylidene-capped (c) and uncapped (d). The bond length and the bond length alternation (BLA) as a function of the number of carbon atoms comprising the wire are reported in panels (e) and (f), respectively. The data are from DFT calculations.

Beginning with the structural properties, the most significant parameter in this context is the bond length alternation (BLA). The BLA is the difference between the average length of quasi-single and quasi-triple bonds in the chain. It is well-known that an increase in the length of the sp (or sp^2^) carbon chain induces an increase in the π-electron conjugation, corresponding to a decrease in the BLA [26,28,36–39], which can be easily rationalized by a straightforward application of the Hückel method. Therefore, longer chains will tend to have an even more equalized structure with a smaller BLA [27,30–31,39–42], even if the occurrence of Peierls distortion would make the perfectly equalized chain unstable. Given this, the markedly polyynic structure found in most of the synthetized sp-carbon chains is usually related to the influence of Peierls distortion. Indeed, the BLA decreases with increasing chain length (π-conjugation) for both polyynes and cumulenes (see Figure 2), similar to many other polyconjugated materials. In contrast, a detailed computational analysis on long sp-carbon chains [42] clearly demonstrated that Peierls distortion dominates over the decrease of the BLA caused by the increasing degree of π-electron delocalization only in relatively long chains. It was verified that C*_N_* chains possess a cumulenic structure determined by end effects for *N* < 52, while in longer chains, the onset of Peierls distortion imposes the alternating structure. This is consistent with the fact that Peierls distortion may be rigorously defined only for an infinite chain. Hence in shorter sp chains, the presence of end effects cannot be overlooked [41–42] and the structure (BLA) of finite sp chains is determined mainly by the chemical nature of the end-capping groups. In Figure 2, the BLA and C–C bond lengths (computed with DFT) are reported for a selection of differently capped sp chains [40]. In hydrogen-capped chains, the H-terminal forces the formation of a triple bond on the adjacent C–C bond, thus a single bond is formed on the next C–C bond, inducing a polyynic structure. A vinylidene cap (i.e., =CH_2_) induces a C=C bond on the sp-chain end, thus promoting a much more equalized, cumulene-like, structure. For the same number of C atoms in the sp chain, the vinylidene-capped chains exhibit a much more equalized structure with respect to hydrogen-capped chains, highlighting that the geometry of finite sp carbon chains is not dictated by Peierls distortion but is completely due to end effects. This property paves the way for the design of new sp-carbon compounds where, by a proper choice of the end groups, it is possible to modify the chain structure, possibly down to very low BLAs, thus forming structures that can be practically considered as cumulenic. By consequence, the electronic properties of the conjugated system can be modulated from a semiconducting to a metallic behavior. On these grounds, the recent works by Tykwinski et al. [43–44] are particularly useful. Long sp-carbon chains, containing up to nine cumulated C=C bonds were synthesized by a proper choice of the end-capping groups. They represent, to the best of the authors’ knowledge, the first long cumulenes obtained by rational chemical synthesis.

### Synthesis techniques and stabilization strategies

Various physical and chemical techniques can be used to produce sp carbon wires in several forms, mainly by bottom-up approaches [5]. Physical techniques are mostly based on the rapid quenching of a carbon vapor in various environments. Supersonic carbon cluster sources based on the arc discharge between graphite electrodes (i.e., the pulsed microplasma cluster source (PMCS) developed by Milani and co-workers) resulted in sp–sp^2^ hybrid amorphous carbon films with an estimated sp content up to 40% [45–46]. Unfortunately, the sp phase easily undergoes rearrangement to the sp^2^ phase when the sample is exposed to air due to oxidative and cross-linking effects and thus requires in situ characterization techniques, as reported in many papers [7,47]. A similar approach was exploited using thermal or laser vaporization cluster sources [6,48]. sp carbon has also been produced by ion irradiation of amorphous carbon [49] and by femtosecond (fs) laser irradiation of a graphite target [50]. fs laser pulses were used to produce amorphous carbon films containing sp, sp^2^ and sp^3^ fractions, however control over their relative quantities was not demonstrated [51]. Isolated wires can be produced by laser ablation (with both fs and ns pulses) of carbon solid targets or suspensions in liquids and particularly in the polyyne-like form with an even number of carbon atoms [7,46]. With reference to polyynes in solution, an easy-to-use and cost-effective technique is the arc discharge in liquids developed by Cataldo [52]. This technique also allows for the control of the chain termination by selecting suitable solvents [53].

Various chemical techniques have been used to synthesize a large number of sp carbon chains terminated with different molecular groups as reported in the review by Szafert and Gladysz [54]. Among the most commonly employed chemical routes are: dehydropolycondensation of acetylene, the Glaser reaction based on the oxidative coupling reaction of ethynyl groups by copper salts, polycondensation reactions of halides, and dehydrohalogenation of polymers such as the chemical carbonization of poly(vinylidene halides) (PVDH). Wire formation via self-assembly of carbon atoms in the presence of Pt atoms on graphene has been recently reported by Kano et al. [55].

With reference to top-down methods, the only technique proposed so far is electron bombardment, sometimes even accompanied by application of axial stress, in systems such as carbon nanotubes [56] or a single graphene flake. The electron beam of a TEM allows the selective removal of carbon atoms until a single atomic chain is formed as a junction between nanotubes or connecting two separate graphene edges [13]. Other systems of carbon wires connected to graphene edges have also been reported by some authors [57–59].

One of the major problems arising during the synthesis of sp-carbon wires is the stability of the structures. A viable route for the synthesis of stable structures is the stabilization of preformed wires and a few attempts have been made in this direction so far. We demonstrated that H-terminated polyynes could be embedded in a solid assembly of Ag nanoparticles resulting in a sample which is stable for several weeks at room temperature under ambient conditions [60]. Hayashi and co-workers showed that it is possible to produce a polymeric composite (i.e., poly(vinyl alcohol)) containing polyynes stabilized by Ag nanoparticles [61].

Due to their high stability, polyynes in liquids (up to 14–16 carbon atoms) can now be synthesized even in the form of size-selected samples [5,62–63] and with well-defined end groups [54]. Solid-state samples have been also produced in powder form [64] and Chalifoux and Tykwinski recently reported the synthesis of chains of up to 44 carbon atoms terminated by bulky groups [65]. The latter system is stable in air and at room temperature in the form of a solid sample. On the contrary, cumulenes seem more difficult to produce and fewer works report their observation. For instance, both cumulenes and polyynes have been detected in a pure sp–sp^2^ cluster-assembled system [9,66] and the higher tendency of cumulenes to undergo sp^2^ transformation has been outlined [45,67–68]. By modification of the termination-induced electronic arrangement, short cumulenic structures can be produced, as reported by Cataldo [69] and extensively discussed in the review by Cadierno et al. [70]. A significant step in the preparation of long cumulenic chains was very recently presented by the group of Tykwinski [43–44]: by adopting different synthesis procedures, long cumulenes chains (up to 8 sp-carbon atoms) have been selectively obtained. Again, the end caps play a fundamental role for two reasons. The first is that due to their chemical nature, they promote the formation of a double bond on the first bond of the sp chain, as required to induce a cumulenic structure. Secondly, they are chosen to be bulky enough to prevent interactions among sp chains, thus avoiding cross-linking and degradation.

### Raman spectroscopy of carbon-atom wires

As pointed out when discussing the structural and electronic properties, the vibrational properties (notably Raman activity [71–72]) of CAWs are similar to other one-dimensional, polyconjugated carbon systems, such as polyacetylene and polyenes. The Raman spectra of these π-electron systems have been extensively investigated [26,28,36–38] and show a peculiar behavior. In particular, the dominant feature originates from the oscillation of the BLA which is an out-of-phase C–C stretching and is named the “R mode” or “ECC mode”, according to the effective conjugation coordinate (ECC) model [26,28,36–37]. The spectral peak shifts to a lower wavenumber and with an increased intensity for an increasing wire length (i.e., number of carbon atoms). In addition, recent theoretical analysis carried out with DFT suggests that for long wires under axial strain along the sp-chain, anharmonicity may also drastically affect the Raman spectra, resulting in an interesting interplay with Peierls distortion effects [73].

**Figure 3:**
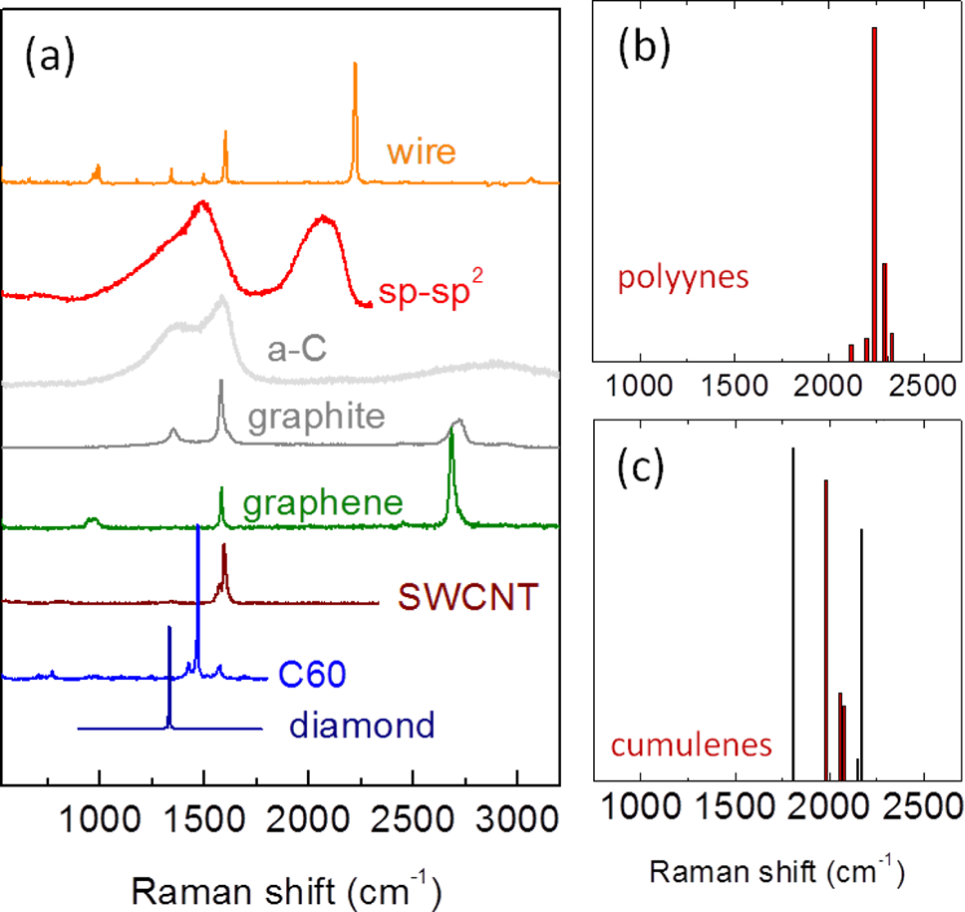
(a) Experimental Raman spectra of carbon solids and nanostructures. (b,c) DFT-computed Raman peaks for finite polyynes and cumulenes of different lengths. Figure adapted with permission from [40], copyright 2010 John Wiley & Sons.

Examples of the extreme sensitivity of Raman spectroscopy to the carbon hybridization state, electronic structure and local order, are shown in Figure 3, where different carbon systems are characterized by well-defined Raman scattering features. In contrast to the other forms of carbon (e.g., fullerenes, nanotubes, graphene), the Raman spectra of sp-carbon chains has been only recently investigated in detail, and a consistent description has just begun to emerge. The Raman spectrum of polyynes shows a similar behavior to polyenes with a very intense feature named the “α line” by some authors [62] which corresponds to the ECC mode. In addition a second, minor band (β line) is often observed. These fingerprints lie in the 1800–2300 cm^−1^ region and they are related to different collective stretching vibrations of sp-hybridized C–C bonds (i.e., BLA oscillation modes), which have been discussed in detail in [32] through theoretical analysis and first-principles calculations. This spectral region is particular to sp carbon, since none of the other carbon nanostructures have peaks in this region (see Figure 3). Within this spectral region cumulenes exhibit lower overall Raman signals than polyynes. DFT calculations have quantitatively shown that for realistic systems of finite length, the strong electron–phonon coupling is responsible for the red-shift of the Raman mode when increasing the length [31,33,35,41].

Furthermore, a first interpretation has been carried out by taking into account the infinite chain model, which allows interpretation of the α and β lines on the basis of the longitudinal optical (LO) phonon dispersion branches of an infinite, Peierls-distorted, carbon chain (i.e., with two atoms in the unit cell). Similar to the tendency of the electronic structure (where the band gap is modulated by the BLA, showing a transition from semiconducting (BLA ≠ 0) to metallic states (BLA = 0)), the LO phonon branch is also strongly modulated by the BLA and shows a Kohn anomaly at Γ in the case of a cumulenic chain [30–33,35]. This behavior can be interpreted on the basis of the modulation of the ECC mode force constant, driven by the occurrence of increasingly more π-electron delocalization with decreasing BLA. This point has been theoretically demonstrated by means of the Hückel model [29,32–33], which highlights the important role of long-range vibrational interactions among C–C stretching coordinates in the sp-carbon chain. As in the case of standard polymer systems, the wavenumbers associated with LO vibrations in finite length chains can be correlated to the LO branch of the respective infinite model at different points of the first Brillouin zone. This procedure, reported elsewhere in detail [33,72], consists of analyzing the displacement vectors associated with C–C stretching normal modes, finding the nodal pattern and associating the corresponding phonon wavevector. For polyynes, the ECC modes of a finite chain characterized by a given BLA can be correlated to the LO dispersion branch obtained for the same BLA value. This ideal model can be successfully adopted to give an interpretation of the Raman spectra of hydrogen-capped chains of increasing length [30–31] and long polyynes (containing up to 20 conjugated triple bonds) capped with bulky groups [72]. The same approach also allows the Raman spectra of cumulenic species to be modeled [40]. The latter case is particularly interesting since it highlights the inherent weakness of the infinite chain model. The possibility of detecting cumulenic chains by Raman spectroscopy has been often challenged based on the fact that an infinite chain with equivalent double bonds would be a monoatomic chain with no optical phonon branch. However, the existence of cumulenic molecular systems has been revealed by Raman spectroscopy for mixed sp/sp^2^ carbon nanostructures [9,47,66]. This apparent contradiction can be solved by relaxing the infinite chain model. While only phonons at Γ have non-negligible Raman activity for an infinite polymer, for finite chains, vibrations located on the LO branch at different points of the 1st Brillouin zone can also be Raman active, due to end effects (which are obviously lacking when periodic boundary conditions are assumed). For instance, the Raman spectra of several DFT-computed cumulenic (C*_N_*) chains are reported in [40]. Many vibrational transitions show non-negligible Raman intensity, thus demonstrating the possibility of detecting cumulenic chains by means of Raman spectroscopy. Interestingly, the interplay between the activation of out-of-Γ normal modes and the molecular parameters governing Raman activity [29,74] generates a detectable Raman signal for LO modes other than ECC in cumulenes, as shown in Figure 3(b,c). In reference to cumulenes, it has to be observed by Liu et al. that finite cumulenes have a well-defined torsional stiffness. Therefore, the relative twisting vibrations of the CH_2_ end groups should be considered as potential Raman signals useful for the characterization of these systems, given that their respective force constant decreases with 1/length [75]. However, in this respect, calculations of the Raman response of these modes [40] have revealed that such modes are expected to be very weak (or inactive) compared to the bending modes which occur in a close wavenumber range and have been observed in [67], thus hindering the detection of these CH_2_ twisting modes.

Based on the above discussion, it should be clear how end effects might affect the overall properties of sp-carbon chains, not only by modifying the molecular structure (i.e., BLA) but also by influencing the electronic and vibrational properties. By properly choosing the end groups, one can modulate the structure of the chain with the aim of modulating the band gap of the system. This effect can be spectroscopically probed due to the evolution of the distinct marker bands observed in the Raman spectra. Hence, Raman spectroscopy, enhanced by the strong predictive power of first-principles simulations, constitutes a powerful, non-invasive characterization technique, which can provide valuable information on the molecular properties of sp-carbon systems.

We now introduce a few case studies where Raman spectroscopy proved to be particularly insightful for the characterization of sp-carbon systems. For two cases (hydrogen- and phenyl-terminated polyynes) we will show that Raman spectroscopy allows the identification of CAWs of different lengths. Furthermore, by comparing Raman and SERS we will discuss the occurrence of charge transfer between CAWs and metal nanoparticles used as the SERS active medium. Such charge transfer results in a change of the electronic configuration of the wire that evolves towards a more equalized structure (i.e., cumulenic).

**Figure 4:**
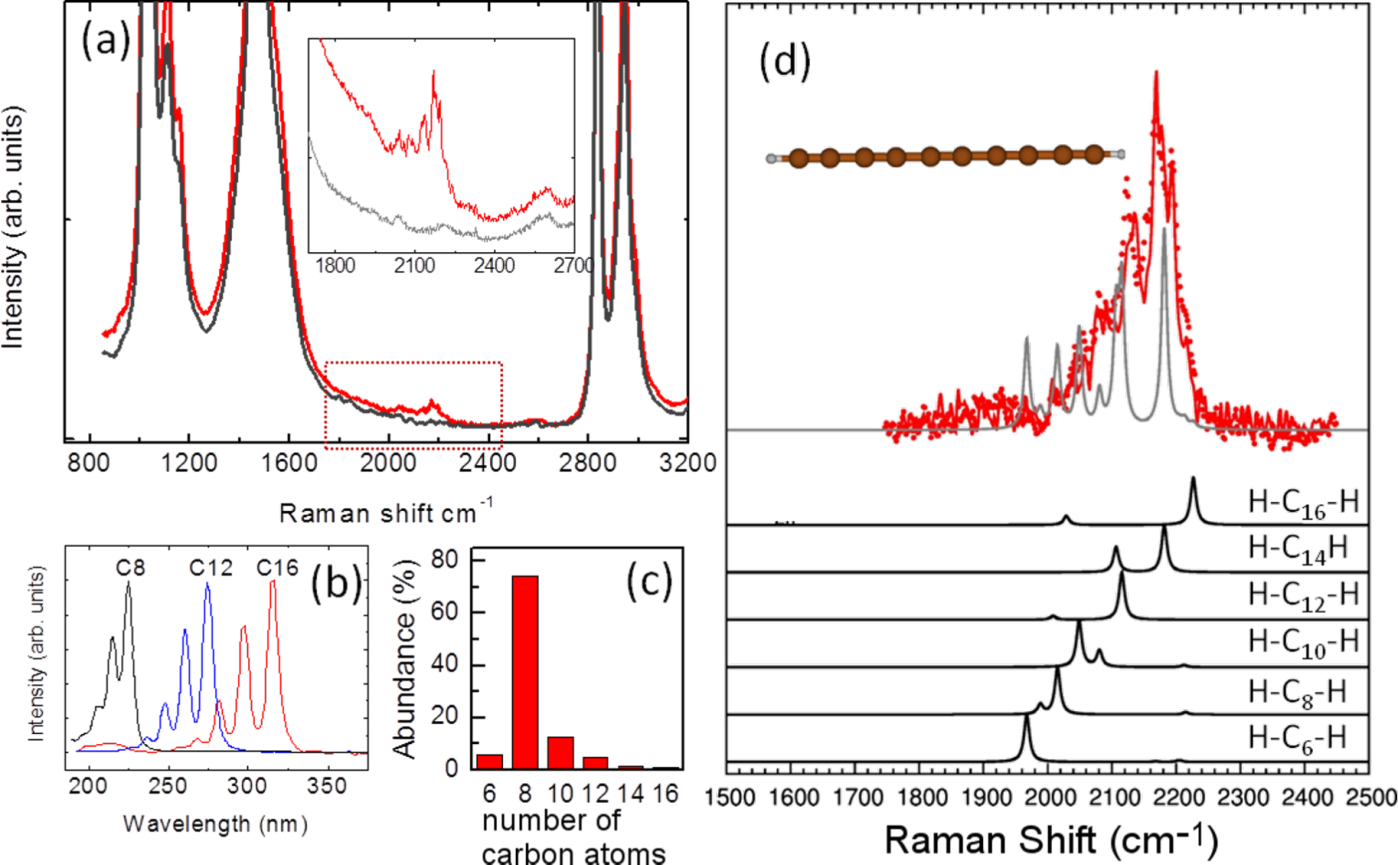
(a) Experimental Raman spectrum (1064 nm) of H-capped polyynes in methanol (5 × 10^−3^ M), with the pure solvent shown for comparison. (b,c) UV–vis spectra for polyynes of different lengths and size distribution in the sample solution. (d) DFT-computed Raman peaks for polyynes present in the sample weighted by their correspondent abundance and comparison with the experimental spectrum. Figure adapted with permission from [76], copyright 2006 Elsevier.

H-terminated polyynes were produced by the submerged arc discharge technique, as described in detail in [64]. When the discharge is operated in methanol it is possible to obtain polyynes with an even number of carbon atoms (6 ≤ C ≤ 16) terminated by one hydrogen atom on each side. The size distribution obtained from HPLC complemented with UV–vis spectroscopy is reported in Figure 4. Even at low concentration it is possible to obtain a Raman spectrum from the liquid sample as shown in Figure 4a, where the low intensity of the sp signal is clear when compared to the signal of the solvent (methanol). The sp signal consititutes an asymmetrical band extending from 2000 cm^−1^ to 2250 cm^−1^ as a result of the contribution of wires of different lengths.

**Figure 5:**
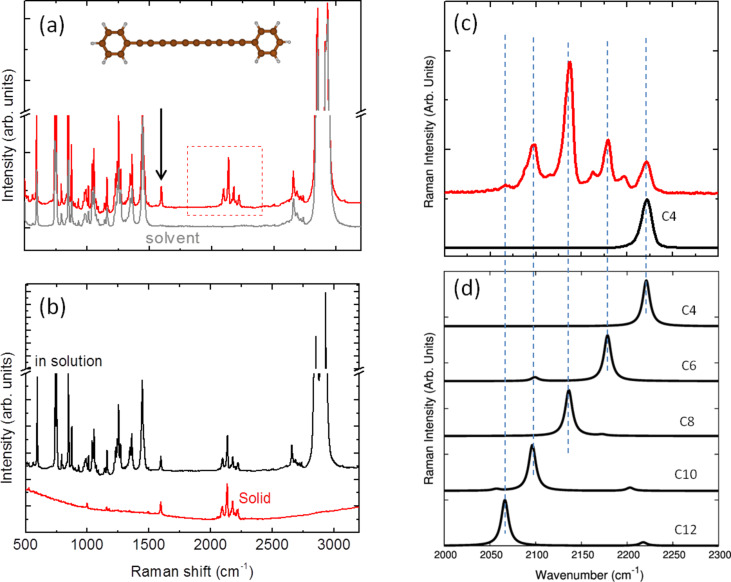
(a) Experimental Raman spectrum (1064 nm) of phenyl-capped polyynes in decalin (10^−2^ M) with the pure solvent shown for comparison. The peak related to the phenyl termination is marked with an arrow. (b) The Raman spectrum of phenyl-capped polyynes after solvent removal to show the stability of the sample. (c) Detail of the experimental Raman spectrum of panel (a). The spectrum of size-selected phenyl-capped polyyne with 4 carbon atoms is also reported. (d) Peaks corresponding to different wire lengths according to DFT calculations of the Raman modes [39]. Figure adapted with permission from [39], copyright 2011 American Chemical Society.

As previously mentioned the vibrational features are strongly dependent on the wire length which is clearly shown in the theoretical spectrum obtained by computing the active Raman modes for single wires with 6–18 carbon atoms. The wavenumber of the Raman modes decreases for longer wires while the Raman intensity increases. Although the correct Raman intensity behavior as a function of chain length is not very accurately captured by the DFT calculations, by summing the different contributions and properly weighting by their quantity, we can obtain a fair representation of the experimental spectrum. Hydrogen-terminated polyynes show limited stability with time since they easily undergo a transition towards sp^2^ as a consequence of cross-linking reactions [77]. It is known that end groups bulkier than hydrogen, such as phenyls or even larger caps [65], impart stability to CAWs. Phenyl-terminated polyynes were produced by chemical synthesis with details given in [78]. Due to the termination type, these systems are stable at ambient conditions even when the solvent is completely removed and the sample is in the solid state, as shown in Figure 5. The Raman spectrum of diphenyl-polyynes is characterized by well-resolved peaks in the 2050–2250 cm^−1^ region. Also in this case, this is due to the size distribution of polyynes in the sample and is confirmed by the spectrum of the size-selected sample with 4 carbon atom wires. The additional peak at 1600 cm^−1^ is related to the stretching of the phenyl ring, hence, it is a marker of the termination with sp^2^ character.

DFT calculations of the Raman spectra of several phenyl-capped polyynes of selected length allow the assignment of each observed peak to a given size of the sp chain. In this case, a significant red shift of the ECC mode for increasing chain lengths is also observed, which is consistent with an increase of π-conjugation. The Raman intensities of the computed spectra reported in Figure 5d are normalized to allow a better comparison with the experimental spectra.

**Figure 6:**
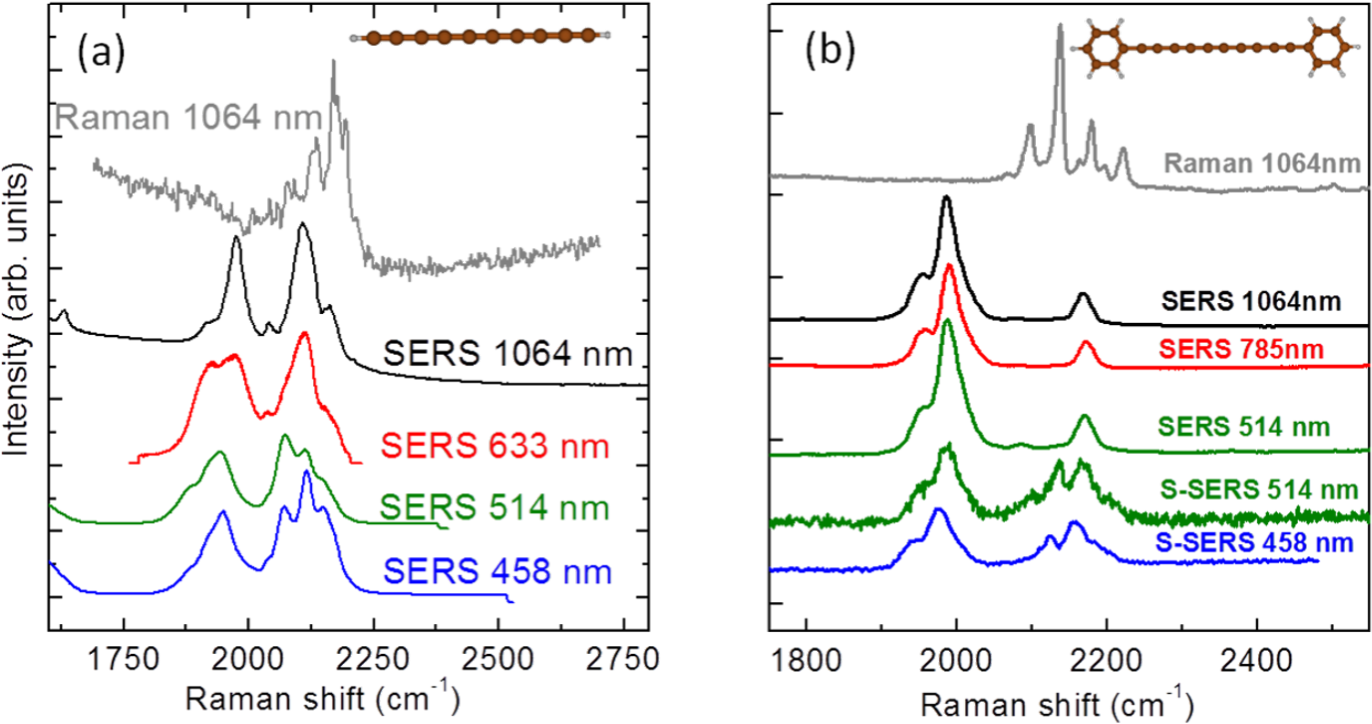
Raman and SERS spectra of H-capped (a) and phenyl-capped (b) polyynes in solution at different excitation wavelengths. SERS on solid surfaces (S-SERS) of phenyl-capped polyynes are also reported. The Raman spectra (at 1064 nm) of both systems are shown for direct comparison. Figure adapted with permission from [39], copyright 2011 American Chemical Society.

A peculiar effect is observed when polyynes (both H- and phenyl-terminated wires) interact with metal nanoparticles (i.e., silver and gold), such as those employed in SERS to increase the sensitivity of the Raman technique. Interaction with metal nanoparticles has been investigated both in solution and on surfaces. SERS in solution has been carried out by adding silver and gold colloids to the sample solution while for surface SERS (S-SERS), silver and gold nanoislands supported on silicon and glass substrates have been used [39]. It was observed that the SERS spectrum is radically different from the Raman spectrum. A shift in the main Raman peak locations is accompanied by the appearance of new spectral features at lower wavenumbers (below 2000 cm^−1^), as shown in Figure 6 for the case of silver nanoparticles. This occurs also in the case of gold nanoparticles for different excitation wavelengths ranging from NIR (1064 nm) to blue (458 nm) wavelengths, illustrating that this is not a resonance-activated effect [39].

When interacting with metal nanoparticles in solution, H-terminated polyynes promote colloid aggregation, which causes the plasmon resonance to broaden and shift from the visible to the NIR. This effectively allows SERS to be performed at different excitation wavelengths. This behavior of H-terminated polyynes can be rationalized by assuming a strong chemical interaction with metal particles or even substitution of the hydrogen with silver. This effect can also justify the increased stability observed after mixing with silver nanoparticles [60]. Phenyl-capped polyynes show similar aggregation when mixed with metal nanoparticles.

**Figure 7:**
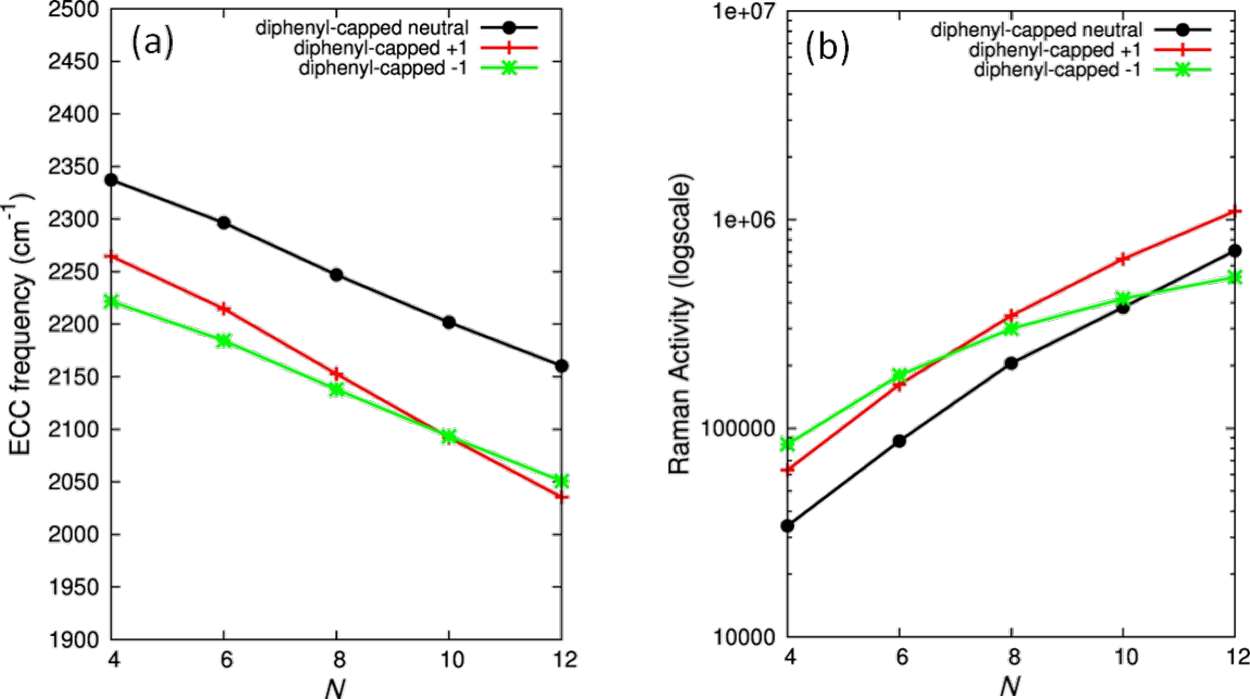
(a) Modulation of the DFT-computed [39] vibrational frequency and (b) Raman activity of the ECC band for phenyl-capped polyynes of different lengths (*N*) and charge states (0, +1, –1). Figure adapted with permission from [39], copyright 2011 American Chemical Society.

The changes occurring in the vibrational properties of wires upon interaction with metal nanoparticles (i.e., observed comparing Raman with SERS) suggest a chemical SERS effect with total enhancement factors up to 10^6^, as observed in the case of H-capped polyynes [76]. In order to explain this observation, the possibility of a charge transfer between the metal and the carbon wire has been proposed [39]. Computing the Raman shift of CAWs of different lengths and comparing neutral CAWs with charged ones, it is straightforward to observe a relevant softening of the ECC Raman modes and an increase of their Raman activity, which is promoted for larger conjugation, as reported in Figure 7.

For instance, for a wire of a given size, a decrease of about 100 cm^−1^ is predicted when the wire is charged, both by adding or removing one electron. The trend in both peak location and Raman intensity is similar even though the shifting effect is slightly different upon oxidation/reduction in long/short chains. By consequence, in both positively or negatively charged diphenyl-capped polyynes, new bands would appear in the spectra at lower wavenumbers and with a larger Raman activity, which can explain the recorded experimental SERS spectra.

**Figure 8:**
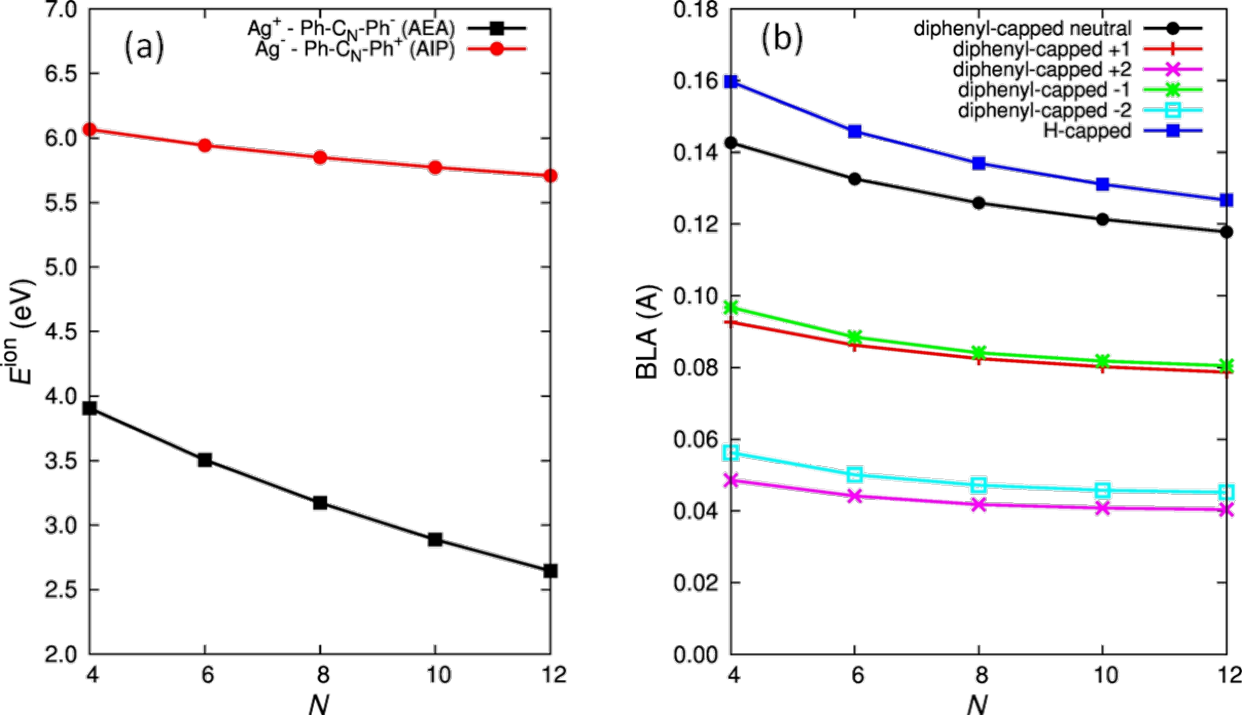
(a) Plot of the DFT-computed energy [39] required for the formation of the charged species (*E*_ion_ = IP(A) − |EA(B)| where IP(A) is the ionization potential of A and EA(B) is the electron affinity of B) for the two possible cases Ag^+^ [Ph–C_N_–Ph]^−^ and Ag^−^ [Ph–C_N_–Ph]^+^ of phenyl-capped polyynes of different chain lengths (*N*). The following experimental values for the work function and EA have been used in the case of Ag: IP = 4.6 eV and EA = −1.30 eV for EA [34] (b) Modulation of the DFT-computed [39] BLA for phenyl-capped polyynes of different lengths (*N*) and charge states (0, +1, −1). Figure adapted with permission from [39], copyright 2011 American Chemical Society.

Figure 8a reports the energy *E*^ion^ required for the formation of the two possible charged configurations for each wire length, namely Ag^+^ [Ph–C*_N_*–Ph]^−^ and Ag^−^ [Ph–C*_N_*–Ph]^+^. Given a pair of ionic species A^+^..B^−^, *E*^ion^ is defined as *E*^ion^ = IP(A) − |EA(B)|, where IP(A) is the ionization potential of A and EA(B) is the electron affinity of B. By evaluating this term for the two charged configurations indicated above, we can determine the direction of the charge transfer since the complex possessing the lower value of *E*^ion^ would be preferentially formed.

IP and EA have been calculated for neutral and charged diphenyl-capped polyynes: in particular “adiabatic” IP (AIP) and EA (AEA) are reported by considering the total energy of the charged species in their minima, thus including geometry-relaxation effects upon charge transfer. For Ag we have considered the experimental values of IP and EA [39].

Based on this calculation and Figure 8a, it is clear that the configuration with a positively charged metal and negatively charged wire is favored. Furthermore, *E*^ion^ is also modulated by π conjugation in this case. For increasing chain lengths (i.e., larger conjugation) the energy required for the formation of charged species decreases, thus favoring the charge transfer process.

In addition, since charge transfer obviously alters the electronic structure of the wire, we can expect some effect also on the molecular structure. This is due to the strong, characteristic electron–phonon coupling existing in π-conjugated systems which connects the electronic effects with the structure of the sp chain. This indeed occurs, as demonstrated by the BLA values computed for the neutral and charged species (Figure 8b). Charge transfer induces a BLA decrease in the polyyne structure, which evolves towards a more equalized structure. In other words, upon charge transfer the wire moves from an alternating (polyyne) to an equalized (cumulene) wire configuration. The reduction amounts to more than 30% for a singly charged wire and more than 60% for a doubly charged wire, reaching a lower value of 0.04 Å for 12 carbon atoms. It is important to notice that for finite-length wires, the ideal cumulene structure with BLA = 0 Å is not realistic due to the influence of the terminations. The end effects are stronger in shorter wires, as shown in Figure 2f where the BLA of finite cumulenes is reported. Vinylidene-capped systems have a BLA which ranges from about 0.05 Å to 0.02 Å moving from 4 to 10 carbon atoms. Uncapped C*_N_* cumulenes show a BLA below 0.02 Å even though they represent a model system that is experimentally unfeasible, except in extreme conditions. As a reference, this result agrees with theoretical calculations by Weimer et al. [79] reporting an increase in the BLA in cumulene wires from 0.006 to 0.048 when the chain length is decreased from 40 to 4 atoms.

This approach gives only a qualitative evaluation of the charge transfer since it does not include the effect of the wire-to-metal interaction in the calculation. The proposed effect is reasonable, although a more complete model should consider the whole wire and metal system and their interaction. This is indeed extremely complex and time consuming from a calculation point of view due to the large numbers of atoms and the need to consider electronic and vibrational properties.

## Conclusion

sp-carbon-atom wires show appealing properties for fundamental studies and applied research. They represent an additional player in the family of carbon nanostructures and can be potentially integrated with graphene and nanotubes to take advantage of their widely tunable electronic and optical properties. We have reviewed the present understanding of structure–property relationship and the use of Raman and SERS for a detailed investigation of wire structure and electronic properties. Although stable polyynes are currently being synthesized, for technological applications, additional work is needed to reveal the properties of wires assembled in thin films. Cumulenes are particularly appealing as a 1D metal. Even though their synthesis remains challenging, novel cumulenic systems have been recently obtained by control of sp-chain capping [43]. Another strategy in this direction is the modification of the structure of polyynes through the control of charge transfer effects.

sp carbon has also attracted interest as a molecular junction, as shown in a number of theoretical investigations and a few experimental works regarding wires connected to graphene and nanotubes. These have shown interesting electronic and transport properties [80–84]. While experimental work is still focused on the synthesis and structural investigation of different CAWs, mixed sp–sp^2^ systems are attracting the attention of the carbon community as hypothetical, novel, hybrid carbon allotropes [85]. Among possible sp–sp^2^ hybrids theoretically predicted, we mention graphyne systems, as outlined by Hirsch [1]. Such structures are 2D carbon layers where sp^2^ rings form a network through sp, linear connections. For some of these systems, peculiar properties are expected such as the existence of Dirac cones in the electronic band structure and extremely high electron mobility [86].
